# Pneumonia — Forgotten no more

**DOI:** 10.15172/pneu.2013.2/264

**Published:** 2013-07-27

**Authors:** Penelope L. Chapman

**Affiliations:** Griffith Health, Griffith University, Gold Coast Campus, Queensland, 4222 Australia

Pneumonia is the leading cause of death in children worldwide and kills an estimated 1.2 million children under the age of five every year; more than AIDS, malaria and tuberculosis combined [1]. Relatively few resources have been committed to addressing the problem of childhood pneumonia, particularly in resource poor settings. Yet effective interventions are available but reach too few children [2]. Less than 20% of children with pneumonia receive antibiotic treatment and only about half receive appropriate medical care [3]. In the adult population, community-acquired pneumonia remains a major reason for admission to hospital, prolonged stay and premature death in developed countries [4]. Low vaccination numbers and a serious lack of awareness about the risks of contracting pneumonia in this age group are also contributing to the increasing incidence of pneumonia in older age [5].

In 2010, the disease previously dubbed the “forgotten killer of children” by the United Nations Children’s Fund (UNICEF) and the World Health Organization (WHO), was made a global health priority and a resolution was passed by the World Health Assembly recognising pneumonia as the world’s leading infectious killer of children [2].

Increasing attention has been placed on childhood pneumonia, primarily since the launch of the first Global Action Plan for Prevention and Control of Pneumonia (GAPP) in 2009. The GAPP has as its core aim, to increase the awareness of pneumonia as a major cause of child death and to spur action to deal more effectively with the problem, by implementing proven interventions for the prevention and treatment of the disease [6]. The Global Coalition Against Child Pneumonia was also established in 2009 to raise awareness about the toll of pneumonia and to advocate for global action to protect against the disease, introducing activities such as World Pneumonia Day [7]. In recent years, the Bill and Melinda Gates Foundation has also been advocating for protection against childhood pneumonia, partnering with the Global Alliance for Vaccines and Immunisation (GAVI), to provide vaccines to children in the world’s poorest countries [8]. Immunisations are contributing to a reduction in childhood deaths from pneumonia in two ways. Firstly, by preventing children from developing *Haemophilus influenzae* type b and pneumococcal infections that cause bacterial pneumonia, and secondly, by preventing infections such as measles and whooping cough that can lead to pneumonia as a result of complications [3].

As the mean age of the population increases however, it is expected that the burden of pneumococcal infection is also likely to increase [5]. Factors such as age, immunosuppression and cardiopulmonary disease are known to increase the risk of pneumonia in community-dwelling elderly people [9]. Less than one-in-three (29%) of the at-risk population have been vaccinated against pneumococcal pneumonia [10]. Most developed countries have recommendations for pneumococcal vaccination, but vaccination rates have remained low as a result of a lack of awareness of increasing risk factors in advancing age [5].

Earlier this year, a new Integrated Global Action Plan for the Prevention and Control of Pneumonia and Diarrhoea was released by the WHO and UNICEF, stating its potential to save up to 2 million extra children every year from deaths caused by pneumonia and diarrhoea [11]. The Action Plan’s targets are significantly higher than previous levels and for the first time since its inception, focus efforts towards activities dedicated to preventing deaths from both pneumonia and diarrhoea. These diseases are often linked in a vicious cycle that exploit weakened immune systems struggling to overcome co-infections and threatened by basic environmental risks, with many of the solutions needed to fight pneumonia and diarrhoea being complementary [11]. Overall, the Action Plan aims for a 75% reduction in the global incidence of severe pneumonia and diarrhoea, along with the virtual elimination of deaths from both diseases by 2025 [12]. This will be partially dependent on the new target of 90% of children worldwide having improved access to healthcare and appropriate antibiotics [12]. Reducing the burden of pneumonia involves risk reduction through holistic means such as proper nutrition, early and exclusive breastfeeding, reducing indoor air pollution, safe drinking water and sanitation, as well as vaccination and improved care seeking and treatment [7, 10]. Current research in older adults has also highlighted the importance of physician recommendations in encouraging patients to be vaccinated and in particular, being more vigilant about patients at risk of pneumococcal infections in order to increase adult vaccination rates [5]. Ultimately, the availability of low-cost, low-tech medications and prompt treatment with a full course of antibiotics can be life-saving [3].

The online journal **pneumonia**, has been an initiative in development for some years. The original concept of the journal was born at the International Symposium on Pneumococci and Pneumococcal Diseases-5 (ISPPD5) held in Alice Springs, Australia, in 2006 and a subsequent Tri-nation (Australia, Papua New Guinea and Indonesia) meeting that was held in Sydney, Australia, in 2009 [13]. All participants expressed their concern at ongoing preventable mortality from pneumonia at a time when the world had the capacity and the technology to prevent pneumonia deaths [6]. To raise the global profile of pneumonia and advance the fight against childhood mortality, there was a general consensus for the establishment of an international forum for pneumonia. To this end, **pneumonia** was launched and is an online, open access journal that provides a platform for bringing together knowledge related to the pathogenesis, treatment and prevention of the disease.

**pneumonia**’s Editorial Board are passionate and dedicated to advancing the fight against childhood mortality due to pneumonia. The journal’s Editorial Board are well represented across the globe from countries including Argentina, Australia, Belgium, Israel, the Netherlands, Papua New Guinea, Spain, South Africa, the United Kingdom and the United States — all combining efforts to raise the global profile of pneumonia. The members of the Board have extensive academic qualifications and are internationally renowned researchers that have published over 7000 papers combined over the course of their careers. As an open access journal, published articles are freely available from the journal’s website which therefore supports a greater global exchange of knowledge. This international collaboration and ease of access is particularly important for researchers and authors in developing countries, as it provides a forum for publishing their research findings without the associated costs of manuscript submission, publication and online access.

In volume 1 of **pneumonia**, papers were published from Malawi, examining oxygen supply and demand in a large teaching hospital setting [14]; Australia, concluding that qPCR analysis of sera has the potential to aid the aetiological diagnosis of pneumonia [15]; and France, which concluded that viral co-infection was observed mainly in patients with severe pneumonia and that RT-PCR substantially improved pathogen detection [16].

In volume 2, papers have been published from Australia, on the threats facing the success of the pneumococcal conjugate vaccine, specifically serotype replacement [17]; the programmatic and financial challenges to vaccine implementation across the globe [18]; and the Republic of Korea, which reviewed the success of a multi-dimensional program organised around World Pneumonia Day [19].

The staff of the Editorial Office of **pneumonia** are responsible for all aspects of the peer-review and publication process. All activities are performed in-house including manuscript receipt and check-in, reviewer and author correspondence, copy and layout editing and electronic publication; systematically tracking every stage of the process along the way.

The staff have also been developing policy and procedure manuals and been highly involved in the long-range planning of the journal, which has meant addressing the effects of the electronic world on publication. Author submission guidelines have been methodically written for ease of manuscript submission and to assist the editorial staff to promptly progress papers through the peer-review process in a timely manner.

**pneumonia** accepts original research articles, case studies, reviews, critical commentaries, correspondence, highlights and news on all aspects of pneumonia and is able to process and publish these free of charge. Although the journal is in its infancy, the site is making impressive progress with worldwide interest. This highlights the significance of the deadly disease and the importance of having an international forum such as **pneumonia**, devoted to raising the global profile of pneumonia. Programs such as the Bill and Melinda Gates Foundation and their partnership with the GAVI Alliance, the WHO and UNICEF Global Action Plan for Pneumonia and Diarrhoea, activities led by the Global Coalition Against Child Pneumonia such as World Pneumonia Day and the introduction of the scientific journal **pneumonia**, are all helping to ensure that pneumonia receives the funding and attention that it deserves and is no longer labelled as the forgotten disease.
